# Spectral Grouping of Nominally *Aspergillus versicolor* Microbial-Collection Deposits by MALDI-TOF MS

**DOI:** 10.3390/microorganisms7080235

**Published:** 2019-08-02

**Authors:** Michael A. Reeve, Thelma S. Caine, Alan G. Buddie

**Affiliations:** Centre for Agriculture and Bioscience International (CABI), Bakeham Lane, Egham, Surrey TW20 9TY, UK

**Keywords:** *Aspergillus versicolor*, subspecies discrimination, spectral grouping, historical taxonomic assignment, historical microbial collections

## Abstract

Historical microbial collections often contain samples that have been deposited over extended time periods, during which accepted taxonomic classification (and also available methods for taxonomic assignment) may have changed considerably. Deposited samples can, therefore, have historical taxonomic assignments (HTAs) that may now be in need of revision, and subdivisions of previously-accepted taxa may also be possible with the aid of current methodologies. One such methodology is matrix-assisted laser-desorption and ionization time-of-flight mass spectrometry (MALDI-TOF MS). Motivated by the high discriminating power of MALDI-TOF MS coupled with the speed and low cost of the method, we have investigated the use of MALDI-TOF MS for spectral grouping of past deposits made to the Centre for Agriculture and Bioscience International (CABI) Genetic Resource Collection under the HTA *Aspergillus versicolor*, a common ascomycete fungus frequently associated with soil and plant material, food spoilage, and damp indoor environments. Despite their common HTA, the 40 deposits analyzed in this study fall into six clear spectral-linkage groups (containing nine, four, four, four, four, and two members, respectively), along with a group of ten spectrally-unique samples. This study demonstrates the clear resolving power of MALDI-TOF MS when applied to samples deposited in historical microbial collections.

## 1. Introduction

Historical microbial collections often contain samples that have been deposited over extended time periods, sometimes many decades. Over this time, accepted taxonomic classification (and also the available methods for taxonomic assignment) may have changed considerably. Prior to the 1990s, common methods for taxonomic assignment of fungi were based predominantly upon microscopy and the analysis of morphological features, often coupled with taxonomic keys. Whilst DNA-based methods have increased considerably in significance since, samples deposited earlier may have historical taxonomic assignments (HTAs) that may now be in need of revision, and the subdivision of previously-accepted taxa may also be possible with the aid of current methodologies. One such methodology is matrix-assisted laser-desorption and ionization time-of-flight mass spectrometry (MALDI-TOF MS), which is rapid and relatively inexpensive, and has found widespread use in the characterization and identification of biological samples.

MALDI-TOF MS exploits the simple yet elegant laser-initiated ‘MALDI’ soft-ionization process [1], which enables the desorption of large proteins into the gas phase without fragmentation. In addition, the MALDI process adds a single positive charge to a significant proportion of the desorbed proteins [2]. This positive charge allows the gas-phase proteins to be accelerated over a short distance by means of an electrical field, thereafter travelling at constant velocity down an evacuated flight tube. As protein times-of-flight to a detector at the end of the flight tube are proportional to the square root of their mass-over-charge ratios [3], a mass spectrum can readily be generated from the biological sample. Whilst MALDI-TOF MS is possible with a wide range of proteins, for the characterization (or identification) of protein-containing samples, highly-expressed and acid-soluble proteins are frequently employed [4]. This fraction of the proteome also contains many ribosomal proteins.

Numerous methods have been developed for MALDI-TOF MS sample-preparation [3,5,6,7,8,9,10,11], ranging from so-called ‘direct-transfer’ protocols (frequently used for the identification of bacteria and yeasts, particularly in clinical diagnostics) to ‘full-extraction’ protocols after Cassagne et al. [7] (which are commonly used for fungal identifications). In addition to the above, Reeve et al. [12] have developed a broadly-applicable yet simple and inexpensive sample-preparation method that lyses microbial cells by immersion in aqueous acetonitrile that also contains trifluoroacetic acid (TFA) to extract acid-soluble proteins, along with near-saturated and inexpensive-grade α-cyano-4-hydroxycinnamic acid (HCCA) matrix. This method can also be applied to insect [12], plant, [12,13], and seed material [14] simply by macerating the biomass of interest in the above reagent. After immersion (or maceration), the resulting matrix-saturated lysate, which also contains the extracted proteins for sample characterization or identification, is simply dried directly onto the MALDI-TOF MS sample plate ready for analysis. Given the high discriminating power of MALDI-TOF MS [12,13,14,15,16] coupled with the speed and low cost of the method [12], we have applied MALDI-TOF MS-based analysis to historical deposits made to the CABI Genetic Resource Collection that all share the HTA *Aspergillus versicolor*.

*Aspergillus versicolor* (Vuillemin) Tiraboschi [17] is an ascomycete fungus commonly associated with soil and plant material [18], food spoilage [19], and damp indoor environments [20,21,22,23], where it can produce a characteristic ‘earthy’ odor. *A. versicolor* also produces the mycotoxin sterigmatocystin [24]. In addition to *A. versicolor*’s environmental occurrence, invasive aspergillosis is also a significant clinical problem in immunocompromised patients [25], and disseminated aspergillosis in dogs has been linked to *A. versicolor* [26]. As reviewed in [27], *Aspergillus* section *Versicolores* (containing the species *A. amoenus*, *A. protuberus*, *A. sydowii*, *A. tabacinus* and *A. versicolor*) was derived from the original *Aspergillus versicolor* group [28] through five historical taxonomic revisions. Using multilocus DNA sequence-based phylogeny, nine new species (*A. austroafricanus*, *A. creber*, *A. cvjetkovicii*, *A. fructus*, *A. jensenii*, *A. puulaauensis*, *A. subversicolor*, *A. tennesseensis* and *A. venenatus*) were described within *Aspergillus* section *Versicolores* [27]. A subsequent multilocus phylogenetic study [29] synonymized section *Versicolores* with section *Nidulantes* and established that *Aspergillus* section *Versicolores* formed a subclade within section *Nidulantes*.

As a rapid and inexpensive complement to methodologies based upon morphological analysis of growth in culture [29] and nucleic-acid analysis [27,29,30,31,32], in the current article, we have investigated the use of MALDI-TOF MS for spectral grouping of past deposits made to the CABI Genetic Resource Collection under the HTA *A. versicolor*.

## 2. Materials and Methods

The following 40 strains used in this study (Table 1) were obtained from the CABI Genetic Resources Collection, a recognized microbial repository, an International Depositary Authority under the Budapest Treaty, and part of the global World Federation for Culture Collections network of public-service culture collections providing authenticated microorganism and reference material to the scientific community.

All cultures were then grown for 3 days at 25 °C on duplicate Potato Dextrose Agar (Oxoid, Thermo Fisher Scientific, Waltham, MA, USA) plates. ≥99.8% ethanol, ≥ 98% (TLC-grade) α-cyano-4-hydroxycinnamic acid (HCCA) matrix, LC-MS-grade acetonitrile, and 99% ReagentPlus^®^-grade TFA were purchased from Sigma (Gillingham, UK). CHROMASOLV^TM^ LC-MS-grade water was purchased from Fluka (Loughborough, UK).

Fungal biomass was mixed with 60 µL of MALDI reagent 1 (11 mg/mL HCCA matrix in 65% (v/v) acetonitrile, 2.5% (v/v) TFA, and 32.5% (v/v) water) using a plastic inoculating loop coated in biomass. Cell lysis and acid-soluble-protein extraction were carried out at room temperature (20 °C), and samples were left for at least one minute before further processing. One microliter of the resulting crude lysates was then pipetted onto the Bruker sample plate, air dried, and loaded into the spectrometer.

Mass spectrometry covering the mass range between 2 kDa and 20 kDa was carried out using a Bruker Microflex LT linear-mode instrument running the MALDI Biotyper 4.0 applications (Bruker Daltonik, Bremen, Germany) as described in Reeve and Seehausen [15]. All spectra are shown baseline-subtracted, smoothed, y-axis-autoscaled, and covering the mass range 2 kDa to 20 kDa (with x-axis scale increments of 2 kDa). Calibration was carried out using the manufacturer’s ‘BTS’ controls (*E. coli* proteins supplemented with ribonuclease A and myoglobin), using peaks with masses at 3,637.8; 5,096.8; 5,381.4; 6,255.4; 7,274.5; 10,300.2; 13,683.2, and 16,952.3 for calibration according to the manufacturer’s instructions.

Sample preparations from plate-1 and plate-2 replicates were carried out as described above, from which a database of 40 plate-1 reference spectra was generated. For spectral comparison, plate-2 test samples were compared against the database of plate-1 reference spectra and Bruker identification scores were generated as described in Reeve and Seehausen [15]. In these molecular-weight-based spectral comparisons, Bruker identification scores were derived using the standard Bruker algorithm. This first converts raw mass spectra into peak lists, which are then compared between spectra. Three separate values are computed: the number of peaks in the reference spectrum that have a closely-matching partner in the test spectrum (value range 0–1), the number of peaks in the test spectrum that have a closely-matching partner in the reference spectrum (value range 0–1), and the peak-height symmetry of the matching peaks (value range 0–1). The above three values are multiplied together and normalized to 1000, and the base-10 logarithm is then taken to give the final Bruker score (range 0–3). Bruker scores of scores between 2.3 and 3.0 indicate very close relatedness, scores between 2.0 and 2.3 indicate close relatedness, and scores below 1.7 indicate low relatedness.

Methods used to undertake morphological identification were based on Klich [33]. Cultures were recovered from preserved stock and three-point inoculations were prepared on 90 mm plates of Czapek Yeast Autolysate Extract Agar (CYA formulation according to Samson and Pitt [34]). The cultures were incubated in darkness at 25 °C for 7 days. Growth rate was then measured and colony colors (upperside and reverse) were recorded. Using a Nikon D40 camera with a DX Nikkor 18–55 mm f/3.5–5.6 G ED II lens and zoom setting at 45, photographs were taken of the top and base of the 7-day culture plates.

Microscopic examination was performed by removing a small quantity of material from the 7-day plates using a sterile needle, mounting on a glass slide in a drop of lactofuchsin stain (0.2 g acid-fuschin, 50 mL glycerol, and 150 mL lactic acid), adding a cover slip, and examining structures at 400× using an Olympus BH-2 microscope. From the features observed, including vesicle diameter and shape, presence/absence of metulae, colony diameter and size, shape, color, and ornamentation of conidia, the taxonomic key to the *Aspergillus* species [33] was used to determine provisional morphological identification.

## 3. Results

**Figure 1:**
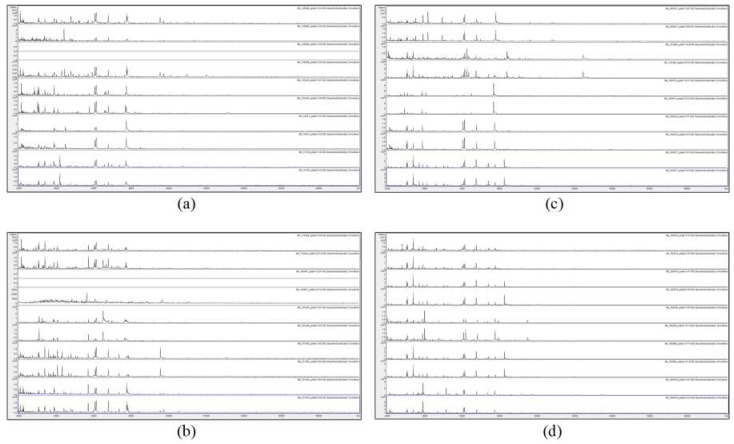
Matrix-assisted laser-desorption and ionization time-of-flight mass spectrometry (MALDI-TOF MS) spectra of acid-soluble fungal proteins from duplicate plates (plate 1 above and plate 2 below), for (**a**) from top to bottom IMI 129488, IMI 129489, IMI 133245, IMI 16041 ii, and IMI 16139; (**b**) from top to bottom IMI 174454, IMI 194967, IMI 210448, IMI 211385, and IMI 211400; (**c**) from top to bottom IMI 226507, IMI 314386, IMI 339610, IMI 349032, and IMI 360877; and (**d**) from top to bottom IMI 360878, IMI 360879, IMI 366228, IMI 360880, and IMI 369918. IMI: Imperial Mycological Institute.

**Figure 2:**
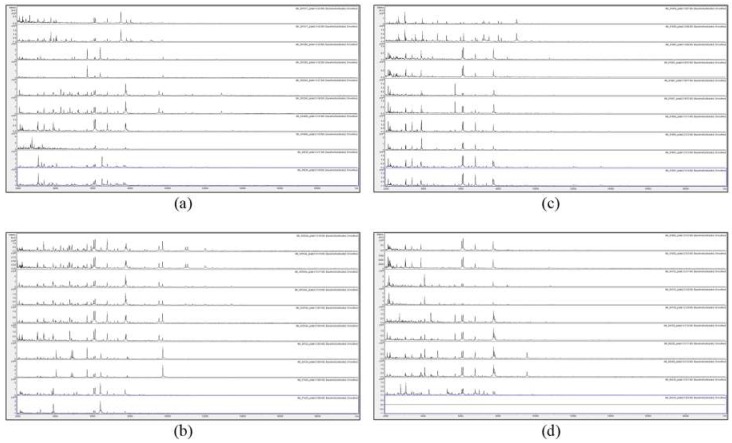
MALDI-TOF MS spectra of acid-soluble fungal proteins from duplicate plates (plate 1 above and plate 2 below), for (**a**) from top to bottom IMI 381617, IMI 381685, IMI 383240, IMI 40496b, and IMI 40636; (**b**) from top to bottom IMI 45554 ii, IMI 45554 iii, IMI, 45554 iv, IMI 49124, and IMI 57426; (**c**) from top to bottom IMI 91859, IMI 91883, IMI 91887, IMI 91890, and IMI 91891; and (**d**) from top to bottom IMI 91892, IMI 94152, IMI 94159, IMI 96228, and IMI 96330.

Figure 1 and Figure 2 show the MALDI-TOF MS spectra of acid-soluble fungal proteins from duplicate plates for all strains with the HTA *A. versicolor* listed in the methods section.

Figure 1 and Figure 2 show that no spectra were obtained from IMI 129489 (plate 1), IMI 194967 (plate 1), and IMI 96330 (plate 2), and poor-quality spectra were obtained from IMI 129488 (plate 2) and IMI 194967 (plate 2) (not included for further analysis). For the remaining 75 samples (94%), peak-rich spectra with good duplication were obtained. Despite the fact that the HTA for each sample is nominally the same (*A. versicolor*), there are visible differences apparent between many of the spectra. In order to discriminate at high resolution between the samples, pairwise spectral comparisons were made. Table 2 shows the Bruker scores generated from spectral comparisons between plate-1 reference-sample spectra and plate-2 test-sample spectra, showing all Bruker scores of 2.0 or greater obtained for each test sample unless the highest score was below 2.0, in which case the highest score obtained is shown (indicated in parentheses). Bruker scores of between 2.300 and 3.000 indicate very close relatedness (‘highly-probable species-level identification’), scores between 2.000 and 2.299 indicate close relatedness (‘secure genus-level identification and probable species-level identification’), scores between 1.700 and 1.999 indicate intermediate relatedness (‘probable genus-level identification’), and scores below 1.699 indicate low relatedness (‘no reliable identification’).

From the 100 spectral comparisons shown Table 2, one score of zero was obtained (because IMI 96330 plate 2 failed to generate a spectrum), and three comparisons were obtained with Bruker scores falling below 2.0 (samples 4, 7, and 24). For the remaining 96 comparisons, Bruker scores exceeding 2.0 were obtained, with an average score of 2.319 and a standard deviation of 0.193.

The data in Table 2 enable the construction of spectral-linkage groups (SLGs), within which all members are related by one or more spectral comparison in Table 2 with a Bruker score exceeding 2.0, and between which SLGs no members have a spectral comparison in Table 2 with a Bruker score exceeding 2.0. Six SLGs are apparent from the data in Table 2.

SLG 1 contains 37 high-scoring spectral linkages [(IMI 383240–IMI 45554ii), (IMI 45554ii–IMI 383240), (IMI 129489–IMI 45554ii), (IMI 129489–IMI 383240), (IMI 383240–IMI 96228), (IMI 45554iii–IMI 383240), (IMI 94159–IMI 96228), (IMI 96228–IMI 129488), (IMI 96228–IMI 45554iii), (IMI 96228–IMI 211400), (IMI 96228–IMI 45554iv), (IMI 96228–IMI 94159), (IMI 96228–IMI 45554ii), (IMI 45554iv–IMI 96228), (IMI 45554iii–IMI 96228), (IMI 45554ii–IMI 96228), (IMI 211400–IMI 96228), (IMI 129489–IMI 96228), (IMI 45554iii–IMI 129488), (IMI 45554iii–IMI 45554iv), (IMI 45554iii–IMI 45554ii), (IMI 45554iv–IMI 45554iii), (IMI 129488–IMI 45554iii), (IMI 129489–IMI 45554iii), (IMI 383240–IMI 45554iii), (IMI 45554ii–IMI 45554iii), (IMI 45554iv–IMI 129488), (IMI 45554ii–IMI 129488), (IMI 383240–IMI 129488), (IMI 129489–IMI 129488), (IMI 45554iv–IMI 45554ii), (IMI 45554iv–IMI 211400), (IMI 383240–IMI 45554iv), (IMI 45554ii–IMI 45554iv), (IMI 129489–IMI 45554iv), (IMI 211400–IMI 94159), and (IMI 94159–IMI 211400)]. SLG 1 members are IMI 129488, IMI 129489, IMI 211400, IMI 383240, IMI 45554ii, IMI 45554iii, IMI 45554iv, IMI 94159, and IMI 96228.

SLG 2 contains 12 high-scoring spectral linkages [(IMI 360877–IMI 360879), (IMI 360878–IMI 360879), (IMI 360879–IMI 360877), (IMI 360879–IMI 360880), (IMI 360879–IMI 360878), (IMI 360880–IMI 360879), (IMI 360877–IMI 360880), (IMI 360877–IMI 360878), (IMI 360878–IMI 360877), (IMI 360878–IMI 360880), (IMI 360880–IMI 360878), and (IMI 360880–IMI 360877)]. SLG 2 members are IMI 360877, IMI 360878, IMI 360879, and IMI 360880.

SLG 3 contains five high-scoring spectral linkages [(IMI 91887–IMI 16139), (IMI 91887–IMI 91891), (IMI 16139–IMI 91891), (IMI 16139–IMI 40496b), and (IMI 91891–IMI 40496b)]. SLG 3 members are IMI 16139, IMI 40496b, IMI 91887, and IMI 91891.

SLG 4 contains four high-scoring spectral linkages [(IMI 40636–IMI 174454), (IMI 40636–IMI 339610), (IMI 40636–IMI 210448), and (IMI 174454–IMI 210448)]. SLG 4 members are IMI 174454, IMI 210448, IMI 339610, and IMI 40636.

SLG 5 contains three high-scoring spectral linkages [(IMI 369918–IMI 91890), (IMI 369918–IMI 57426), and (IMI 369918–IMI 91892)]. SLG 5 members are IMI 57426, IMI 91890, IMI 91892, and IMI 369918.

SLG 6 contains one high-scoring spectral linkage [(IMI 133245–IMI 349032)]. SLG 6 members are IMI 133245 and IMI 349032.

In addition to the above six SLGs, the data in Table 2 show that there are ten spectrally-unique samples (SUSs) that generated no other Bruker score exceeding 2.0 other than the plate-1 against cognate plate-2 comparison. The ten high-scoring SUSs are IMI 211385, IMI 226507, IMI 314386, IMI 366228, IMI 381617, IMI 381685, IMI 49124, IMI 91859, IMI 91883, and IMI 94152 (with plate-1 against cognate plate-2 matches of 2.439, 2.249, 2.289, 2.276, 2.220, 2.361, 2.607, 2.400, 2.021, and 2.130 respectively).

The remaining three samples either failed to generate a plate-2 test spectrum (IMI 96330) or failed to give spectral comparison scores of greater than 2.0 (IMI 16041 ii and IMI 194967).

**Figure 3:**
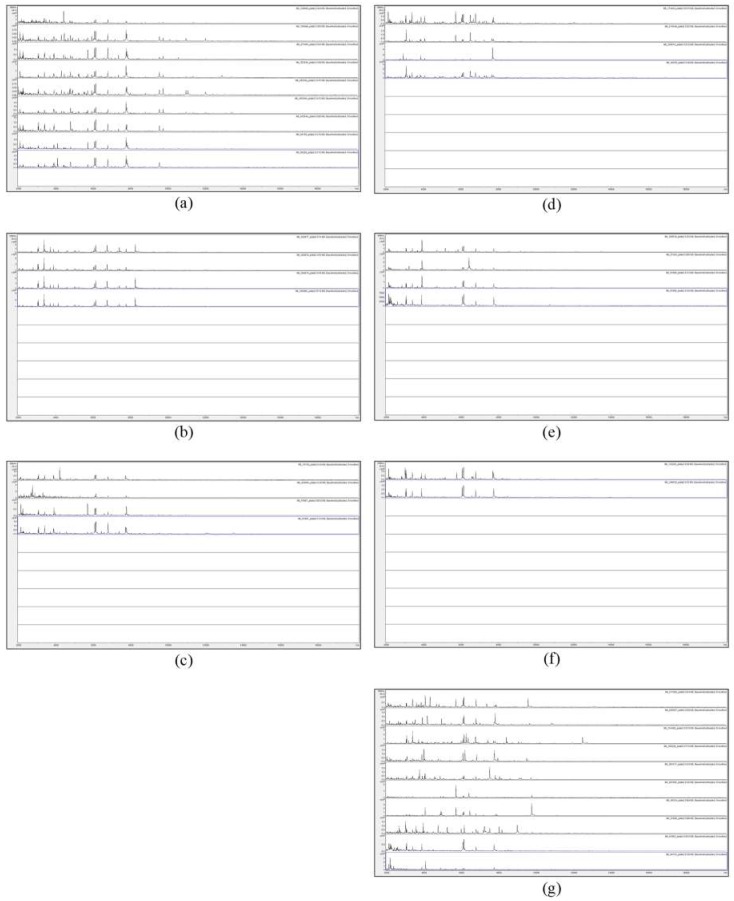
MALDI-TOF MS spectra of acid-soluble fungal proteins from plate-2 samples comprising (**a**) spectral-linkage group (SLG) 1, (**b**) SLG 2, (**c**) SLG 3, (**d**) SLG 4, (**e**) SLG 5, (**f**) SLG 6, and (**g**) spectrally-unique samples (SUSs).

In order to assess visually the spectral consistency within each SLG, Figure 3 shows the MALDI-TOF MS spectra of acid-soluble fungal proteins from the plate-2 samples comprising SLG 1 to SLG 6, along with the ten SUSs.

**Figure 4:**
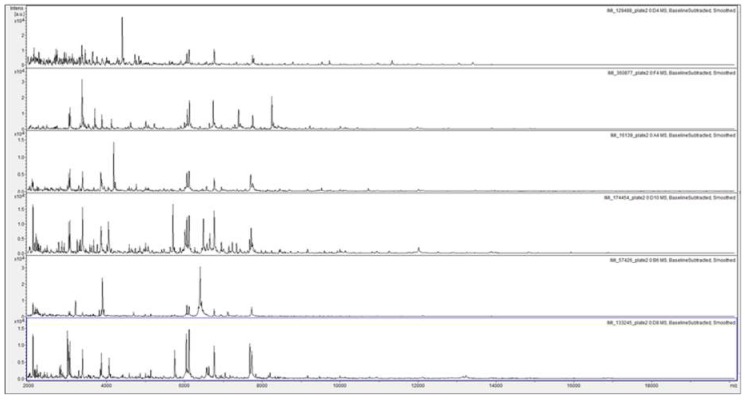
MALDI-TOF MS spectra of acid-soluble fungal proteins from plate-2 examples of, from top to bottom, SLG 1 (IMI 129488), SLG 2 (IMI 360877), SLG 3 (IMI 16139), SLG 4 (IMI174454), SLG 5 (IMI 57426), and SLG 6 (133245).

Figure 3 shows a considerable degree of spectral variation in the SUSs; good consistency between spectra within SLGs 1, 2, 5, and 6; and some variation (mainly due to additional peaks) within SLGs 3 and 4. Figure 4 shows, for ready visual comparison between the six SLGs, MALDI-TOF MS spectra of acid-soluble fungal proteins from plate-2 examples of each of the six SLGs.

Table 3 shows the Bruker scores for spectral comparison against the Bruker database of filamentous fungal samples for the SLGs and SUSs, where Bruker scores between 1.700 and 1.999 (‘probable genus-level identification’) are indicated in parentheses, and Bruker scores below 1.699 (‘no reliable identification’) are indicated by strike-through. The results of independent and blind post-MALDI-TOF MS taxonomic-key-based morphological identifications at seven days (see Figure S1 for example images) are also given for comparison.

## 4. Discussion

Motivated by the high discriminating power of MALDI-TOF MS coupled with the speed and low cost of the method, we have investigated the use of MALDI-TOF MS for spectral grouping of past deposits made to the CABI Genetic Resource Collection under the HTA *A. versicolor*. Despite their common HTA, the 40 deposits analyzed fall into six clear SLGs (SLGs 1, 2, 3, 4, 5, and 6, with nine, four, four, four, four, and two members respectively), along with a group of ten high-scoring SUSs.

Comparison between the spectra obtained and the Bruker database has been carried out but, whilst the grouping of samples on the basis of spectral similarity is clear, the results shown in Table 3 should not be interpreted as definitive taxonomic classifications for the samples analyzed in this study as relatively few species of *Aspergillus* are represented in the Bruker database spectra. Those available in the database are *A. calidoustus*, *A. clavatus*, *A. flavus oryzae* group, *A. fumigatus*, *A. glaucus*, *A. iizukae*, *A. japonicus*, *A. lentulus*, *A. minisclerotigenes*, *A. montvidensis*, *A. nidulans*, *A. niger*, *A. nomius*, *A. ochraceus*, *A. parasiticus*, *A. penicillioides*, *A. pseudoglaucus*, *A. pulvinus*, *A. sclerotiorum*, *A. sydowii*, *A. tamarii*, *A. terreus*, *A. tritici*, *A. unguis*, *A. ustus*, *A. versicolor*, and *A. westerdijkiae*. In addition, spectra found in the Bruker database will have been generated following sample-preparation methods other than the method used in the present study, which may reduce the scorings obtained from spectral comparisons. Bearing these caveats in mind, however, the spectral comparisons do reveal a number of interesting observations.

Firstly, members of SLG 2 show a consistent ‘identification’ of *A. ustus* (supported by taxonomic-key-based morphological identifications) rather than *A. versicolor* as is the case for the remaining five SLGs, with IMI 360877, IMI 360878, IMI 360879 matching most closely to *A. ustus* database-entry DSM 1349 DSM and IMI 360880 matching most closely to *A. ustus* database-entry DSM 63535 DSM. Secondly, whilst SLG 1 is the largest SLG observed in this study, in contrast to any of the other SLGs, the Bruker scores for database entries within SLG 1 all fall below 1.7, suggesting that members of this SLG, whilst closely-related to each other, are spectrally the most remote from any of the Bruker database entries. Thirdly, all members of SLG 4 show a consistent ‘identification’ of *A. versicolor* 2009_137364 MUZ, with higher Bruker scores that suggest closer relatedness of these SLG members to the database entry. Fourthly, higher Bruker scores against database entries are again observed in SLG 6, where the highest ‘identifications’ scores are to *A. versicolor* database-entry F51 LLH. Finally, the SUS IMI 91859 is ‘identified’ as *Penicillium italicum* DSM 2754NT DSM, which is supported by the different growth morphology observed for this strain on the agar plates prior to sampling and by taxonomic-key-based morphological identification. As such, it is clear that this isolate was mis-identified on its deposit to the CABI collection.

It is clear from the above that the HTA given to the 40 strains used in this study (*A. versicolor*) covers a wide range of groupable subtypes from the MALDI-TOF MS spectra resulting from growth in culture followed by a simple and inexpensive method of sample preparation. MALDI-TOF MS, therefore, offers a rapid and inexpensive method for the classification of past deposits made to microbial collections and has great potential alongside complementary methodologies based upon morphological assessment [27,33] and nucleic-acid analysis [27,29,30,31,32] to assist taxonomists working with deposits made under HTAs that now may be in need of further revision or clarification.

## Figures and Tables

**Table 1:** Strains used in the current study.

IMI Number	HTA	Collected From	Collection Location	GPS Coordinates	Notes
16041ii	*A. versicolor*	Manufactured tobacco	United Kingdom	(52.4379°; −1.6496°)	CABI accession 1962
16139	*A. versicolor*		Netherlands		CABI accession 1947, CECT 2903, ATCC 26939
40496b	*A. versicolor*	*Brassica* sp. (dead stem)	United Kingdom	(52.4379°; −1.6496°)	Deposited by SJ Hughes, CABI accession 1950
40636	*A. versicolor*	Paper	Ghana	(8.0000°; −2.0000°)	CABI accession 1950
45554ii	*A. versicolor*	Cellophane paper	Indiana, United States	(40.3363°; −89.0022°)	Deposited by MH Downing, CABI accession 1970, ATCC 11730, CBS 245.65
45554iii	*A. versicolor*	Cellophane paper	Indiana, United States	(40.3363°; −89.0022°)	Deposited by MH Downing, CABI accession 1977, ATCC 11730, CBS 245.65
45554iv	*A. versicolor*	Cellophane paper	Indiana, United States	(40.3363°; −89.0022°)	Deposited by MH Downing, CABI accession 1977, ATCC 11730, CBS 245.65;
49124	*A. versicolor*	Culture contaminant	Njala, Sierra Leone	(8.2333°; −12.0167°)	Deposited by FC Deighton, CABI accession 1952
57426	*A. versicolor*	*Canis lupus* (claw)	United Kingdom	(52.4379°; −1.6496°)	Deposited by PKC Austwick, CABI accession 1954
91859	*A. versicolor*	Paraffin wax			CABI accession 1962
91883	*A. versicolor*	*Nicotiana tabacum*	United Kingdom	(52.4379°; −1.6496°)	CABI accession 1962
91887	*A. versicolor*				CABI accession 1962
91890	*A. versicolor*	Electrical fuse	United Kingdom	(52.4379°; −1.6496°)	CABI accession 1962
91891	*A. versicolor*		United Kingdom	(52.4379°; −1.6496°)	CABI accession 1962
91892	*A. versicolor*		United Kingdom	(52.4379°; −1.6496°)	CABI accession 1962
94152	*A. versicolor*	Cloth	India	(20.0000°; 77.0000°)	Deposited by G Smith, CABI accession 1962
94159	*A. versicolor*	*Gossypium hirsutum*	United Kingdom	(52.4379°; −1.6496°)	Deposited by G Smith, CABI accession 1962
96228	*A. versicolor*	*Poaceae* sp.	United Kingdom	(52.4379°; −1.6496°)	Deposited by ME Lacey, CABI accession 1962
96330	*A. versicolor*	Painted metal amplifier case	United Kingdom	(52.4379°; −1.6496°)	Deposited by J Langham Thompson, CABI accession 1962
129488	*A. versicolor*		United Kingdom	(52.4379°; −1.6496°)	Deposited by M Cole, CABI accession 1967, ATCC 20171
129489	*A. versicolor*		United Kingdom	(52.4379°; −1.6496°)	Deposited by M Cole, CABI accession 1967, ATCC 20172
133245	*A. versicolor*	*Hordeum* sp. (seed)	United Kingdom	(52.4379°; −1.6496°)	Deposited by W Greenaway, CABI accession 1968
174454	*A. versicolor*	Soil	Uttar Pradesh, India	(27.2500°; 80.7500°)	Deposited by JN Rai, CABI accession 1973
194967	*A. versicolor*		Venezuela	(8.0000°; −66.0000°)	Deposited by G Casas, CABI accession 1975
210448	*A. versicolor*	*Vitis vinifera*	Egypt	(27.0000°; 30.0000°)	Deposited by MF Badawy, CABI accession 1976
211385	*A. versicolor*	*Phoenix dactylifera* (fruit)	USA		Deposited by D Bliss, KB Raper and DI Fennell, CABI accession 1977, CBS 584.65, ATCC 16856
211400	*A. versicolor*	*Berberis* sp. (fruit)	Germany	(51.0000°; 9.0000°)	Deposited by M Roberg, KB Raper and DI Fennell, CABI accession 1977, CBS 111.32, ATCC 16845
226507	*A. versicolor*	Soil	Gwalior, India	(20.0000°; 77.0000°)	Deposited by RKS Chauhan, CABI accession 1978
314386	*A. versicolor*	Polyurethane foam			CABI accession 1987
339610	*A. versicolor*	Soil	Egypt	(27.0000°; 30.0000°)	Deposited by AF Moustafa, CABI accession 1990
349032	*A. versicolor*	Mud	West Bengal, India	(24.0000°; 88.0000°)	Deposited by JN Rai, CABI accession 1991, CBS 186.77
360877	*A. versicolor*	Litter	New Caledonia	(−21.5000°; 165.5000°)	Deposited by J Mouchacca, CABI accession 1994
360878	*A. versicolor*	Litter	New Caledonia	(−21.5000°; 165.5000°)	Deposited by J Mouchacca, CABI accession 1994
360879	*A. versicolor*	Litter	New Caledonia	(−21.5000°; 165.5000°)	Deposited by J Mouchacca, CABI accession 1994
360880	*A. versicolor*	Litter	New Caledonia	(−21.5000°; 165.5000°)	Deposited by J Mouchacca, CABI accession 1994
366228	*A. versicolor*	Cheese rind	Switzerland	(47.0000°; 8.0000°)	Deposited by L Petrini, CABI accession 1995
369918	*A. versicolor*				CABI accession, 1996
381617	*A. versicolor*	Soil	Hawaii, United States	(21.1098°; −157.5311°)	
381685	*A. versicolor*	Sand dune	Hawaii, United States	(21.1098°; −157.5311°)	
383240	*A. versicolor*	Mouth wash	Hungary	(47.0000°; 20.0000°)	Deposited by J Varga, CABI accession 2000

Abbreviations used: IMI (Imperial Mycological Institute), CECT (Coleccion Espanola de Cultivos Tipo), ATCC (American Type Culture Collection), and CBS (Centraalbureau voor Schimmelcultures).

**Table 2:** Bruker scores for spectral comparison between plate-1 reference samples and plate-2 test samples.

Sample Number	Test Spectrum	Reference Spectrum	Bruker Score	Sample Number	Test Spectrum	Reference Spectrum	Bruker Score
1	IMI 129488	IMI 45554iii	2.035	23	IMI 383240	IMI 383240	2.708
2	IMI 129489	IMI 129488	2.473			IMI 45554iii	2.435
		IMI 45554iii	2.381			IMI 129488	2.411
		IMI 45554ii	2.337			IMI 45554ii	2.367
		IMI 45554iv	2.306			IMI 45554iv	2.207
		IMI 383240	2.261			IMI 96228	2.110
		IMI 96228	2.160	24	IMI 40496b	IMI 91891	(1.414)
3	IMI 133245	IMI 133245	2.405	25	IMI 40636	IMI 40636	2.575
		IMI 349032	2.059			IMI 174454	2.325
4	IMI 16041 ii	IMI 91883	(1.993)			IMI 339610	2.115
5	IMI 16139	IMI 16139	2.443			IMI 210448	2.018
		IMI 91891	2.324	26	IMI 45554ii	IMI 45554ii	2.613
		IMI 40496b	2.144			IMI 45554iv	2.433
6	IMI 174454	IMI 174454	2.558			IMI 45554iii	2.416
		IMI 210448	2.010			IMI 129488	2.368
7	IMI 194967	IMI 226507	(0.818)			IMI 383240	2.125
8	IMI 210448	IMI 210448	2.190			IMI 96228	2.042
9	IMI 211385	IMI 211385	2.439	27	IMI 45554iii	IMI 45554iii	2.617
10	IMI 211400	IMI 211400	2.385			IMI 129488	2.523
		IMI 96228	2.063			IMI 383240	2.501
		IMI 94159	2.021			IMI 45554iv	2.439
11	IMI 226507	IMI 226507	2.249			IMI 45554ii	2.420
12	IMI 314386	IMI 314386	2.289			IMI 96228	2.129
13	IMI 339610	IMI 339610	2.392	28	IMI 45554iv	IMI 45554iv	2.385
14	IMI 349032	IMI 349032	2.201			IMI 45554iii	2.334
15	IMI 360877	IMI 360877	2.669			IMI 129488	2.303
		IMI 360879	2.595			IMI 45554ii	2.169
		IMI 360880	2.545			IMI 96228	2.138
		IMI 360878	2.470			IMI 211400	2.020
16	IMI 360878	IMI 360878	2.527	29	IMI 49124	IMI 49124	2.607
		IMI 360879	2.455	30	IMI 57426	IMI 57426	2.174
		IMI 360877	2.338	31	IMI 91859	IMI 91859	2.400
		IMI 360880	2.337	32	IMI 91883	IMI 91883	2.021
17	IMI 360879	IMI 360877	2.613	33	IMI 91887	IMI 91887	2.297
		IMI 360880	2.603			IMI 16139	2.076
		IMI 360879	2.577			IMI 91891	2.062
		IMI 360878	2.219	34	IMI 91890	IMI 91890	2.456
18	IMI 366228	IMI 366228	2.276	35	IMI 91891	IMI 91891	2.676
19	IMI 360880	IMI 360877	2.667			IMI 40496b	2.423
		IMI 360879	2.590	36	IMI 91892	IMI 91892	2.111
		IMI 360880	2.573	37	IMI 94152	IMI 94152	2.130
		IMI 360878	2.281	38	IMI 94159	IMI 94159	2.227
20	IMI 369918	IMI 369918	2.557			IMI 211400	2.124
		IMI 57426	2.176			IMI 96228	2.121
		IMI 91892	2.147	39	IMI 96228	IMI 96228	2.483
		IMI 91890	2.059			IMI 129488	2.242
21	IMI 381617	IMI 381617	2.220			IMI 45554iii	2.234
22	IMI 381685	IMI 381685	2.361			IMI 211400	2.203
						IMI 45554iv	2.190
						IMI 94159	2.091
						IMI 45554ii	2.037
				40	IMI 96330	No spectrum obtained	(0)

**Table 3:** Bruker scores for spectral comparison against the Bruker database of filamentous-fungal samples for the SLGs and SUSs, along with taxonomic-key-based identification results.

Sample	Plate-1 Replicate	Bruker Score	Plate-2 Replicate	Bruker Score	Subsequent Key-Based Morphological Identification
SLG 1					
IMI 129488	*A. versicolor* D_16_256_8_1 LLH	1.574	*A. versicolor* D_16_256_8_1 LLH	1.547	*A. versicolor*
IMI 129489	no peaks found	<0	*A. versicolor* 120227_14 ETL	1.508	*A. versicolor*
IMI 211400	*A. versicolor* D_16_256_8_1 LLH	1.688	*A. versicolor* D_16_256_8_1 LLH	1.617	Not identified (no sporulation)
IMI 383240	*Schizophyllum commune* DSM 1026 DSM	1.147	*Lichtheimia corymbifera* J10018851 AUH	1.154	*A. versicolor*
IMI 45554ii	*A. versicolor* D_16_256_8_1 LLH	1.512	*Trichophyton_mentagrophytes_*var_*erinacei* CC6 F29 LLH	1.203	*A. versicolor*
IMI 45554iii	*A. versicolor* D_16_256_8_1 LLH	1.391	*A. versicolor* D_16_256_8_1 LLH	1.331	*A. versicolor*
IMI 45554iv	*A. versicolor* 2009_137364 MUZ	1.371	*A. versicolor* D_16_256_8_1 LLH	1.345	*A. versicolor*
IMI 94159	*A. versicolor* D_16_256_8_1 LLH	1.637	*A. versicolor* D_16_256_8_1 LLH	1.379	*A. versicolor*
IMI 96228	*A. versicolor* D_16_256_8_1 LLH	1.339	*A. versicolor* 2009_137364 MUZ	1.668	*A. versicolor*
SLG 2					
IMI 360877	*A. ustus* DSM 1349 DSM	(1.791)	*A. ustus* DSM 1349 DSM	(1.936)	cf *A. ustus* group
IMI 360878	*A. ustus* DSM 1349 DSM	1.558	*A. ustus* DSM 1349 DSM	(1.734)	*Aspergillus* sp.
IMI 360879	*A. ustus* DSM 1349 DSM	1.607	*A. ustus* DSM 1349 DSM	(1.829)	cf *A. ustus* group
IMI 360880	*A. ustus* DSM 63535 DSM	(1.845)	*A. ustus* DSM 63535 DSM	1.634	cf *A. ustus* group
SLG 3					
IMI 16139	*A. versicolor* F51 LLH	(1.808)	*A. versicolor* DSM 63292 DSM	(1.801)	*A. versicolor*
IMI 40496b	*A. versicolor* F51 LLH	1.582	*A. versicolor* DSM 63292 DSM	1.180	*A versicolor*
IMI 91887	*A. versicolor* F51 LLH	(1.719)	*A. versicolor* F51 LLH	(1.807)	*A. versicolor*
IMI 91891	*A. versicolor* DSM 63292 DSM	(1.838)	*A. versicolor* F51 LLH	1.561	*A. versicolor*
SLG 4					
IMI 174454	*A. versicolor* 2009_137364 MUZ	2.047	*A. versicolor* 2009_137364 MUZ	2.083	*Aspergillus* sp.
IMI 210448	*A. versicolor* 2009_137364 MUZ	(1.742)	*A. versicolor* 2009_137364 MUZ	1.615	*Aspergillus* sp.
IMI 339610	*A. versicolor* 2009_137364 MUZ	(1.903)	*A. versicolor* 2009_137364 MUZ	1.246	*Aspergillus* sp.
IMI 40636	*A. versicolor* 2009_137364 MUZ	(1.826)	*A. versicolor* 2009_137364 MUZ	2.230	*A. versicolor* group
SLG 5					
IMI 57426	*A. versicolor* DSM 63292 DSM	1.502	*A. versicolor* DSM 63292 DSM	1.456	*A. versicolor*
IMI 91890	*A. versicolor* F51 LLH	(1.953)	*A. versicolor* F51 LLH	1.652	*A. versicolor*
IMI 91892	*A. versicolor* F51 LLH	(1.824)	*A. versicolor* F51 LLH	1.561	*A. versicolor*
IMI 369918	*A. versicolor* DSM 63292 DSM	1.557	*A. versicolor* F51 LLH	(1.757)	*A. versicolor*
SLG 6					
IMI 133245	*A. versicolor* DSM 63292 DSM	(1.731)	*A. versicolor* F51 LLH	2.043	*A. versicolor*
IMI 349032	*A. versicolor* F51 LLH	(1.993)	*A. versicolor* F51 LLH	2.110	*A. versicolor*
SUSs					
IMI 211385	*A. versicolor* F51 LLH	1.217	*Fusarium oxysporum* D_16_256_6_5 LLH	1.126	*A. versicolor*
IMI 226507	*A. ustus* DSM 63535 DSM	1.363	*A. ustus* DSM 1349 DSM	1.364	*A. versicolor* group
IMI 314386	*A. ustus* DSM 1349 DSM	1.589	*A. ustus* DSM 1349 DSM	1.547	*A. ustus* or similar
IMI 366228	*Aureobasidium pullulans_*BB DSM 62074 DSM	1.236	*A. unguis* 614 UGB	1.174	*Aspergillus* sp.
IMI 381617	*A. nidulans* DSM 820 BRB	(1.723)	*A. nidulans* 120220_20 PIM	1.720	*A. nidulans*
IMI 381685	*A. versicolor* MPA 1343 MPA	1.088	*A. versicolor* 2008_141783 MUZ	1.020	*A. versicolor*
IMI 49124	*A. versicolor* 2009_137364 MUZ	1.628	*A. versicolor* MPA 1343 MPA	1.309	*A. versicolor*
IMI 91859	*Penicillium italicum* DSM 2754NT DSM	1.657	*Penicillium italicum* DSM 2754NT DSM	1.827	*Penicillium italicum/expansum* group
IMI 91883	*A. versicolor* DSM 63292 DSM	1.545	*A. versicolor* 120227_14 ETL	1.275	*A. versicolor*
IMI 94152	*A. versicolor* F51 LLH	1.515	*A. versicolor* F51 LLH	1.427	*A. versicolor*

## Supplementary Materials

Supplementary materials can be found at https://www.mdpi.com/2076-2607/7/8/235/s1. Figure S1 Examples of morphology from SLGs 1–6 at 7-days on CYA grown at 25 °C.
