# Supplementary material for: Spectral Grouping of Nominally Aspergillus versicolor Microbial-Collection Deposits by MALDI-TOF MS
Source: Microorganisms. 2019 Aug 2;7(8):235. doi: 10.3390/microorganisms7080235 (PMC6722527; doi:10.3390/microorganisms7080235)
Supplement: Supplementary file 1 [file microorganisms-07-00235-s001.pdf]

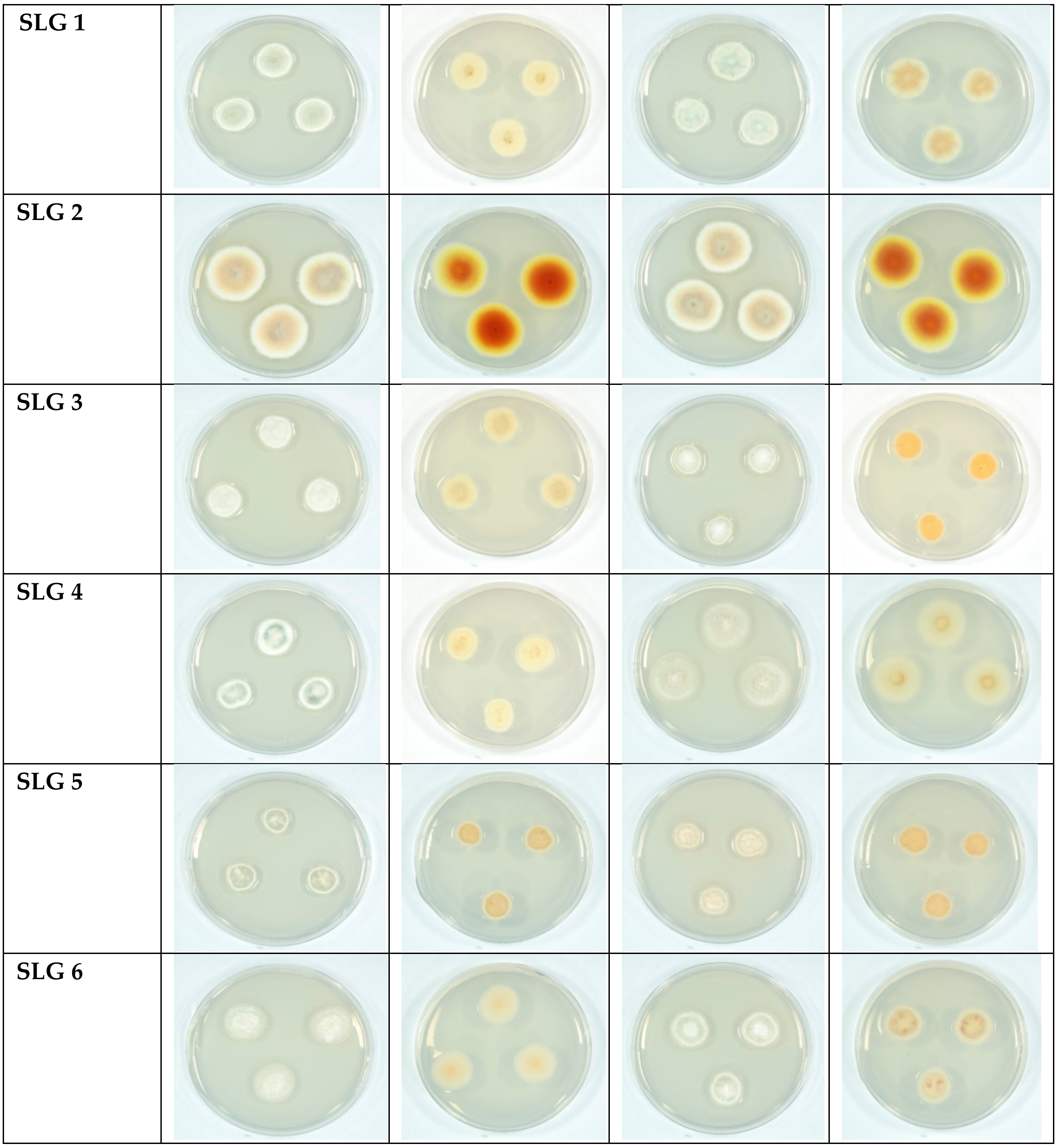

Figure S1. Examples of morphology from SLGs 1-6 at 7-days on CYA grown at 25°C. From top to bottom and left to right: SLG 1: IMI 45554ii (top); IMI 4555ii (base); IMI 94159 (top); IMI 94159 (base); SLG 2: IMI 360877 (top), IMI 360877 (base); IMI 360880 (top); IMI 360880 (base), SLG 3: IMI 16139 (top); IMI 16139 (base); IMI 40496b (top); IMI 40496b (base); SLG 4: IMI 40636 (top); IMI 404636 (base), IMI 339610 (top); IMI 339610 (base); SLG 5: IMI 57426 (top); IMI 57426 (base); IMI 91890 (top); IMI 91890 (base); SLG 6: IMI 133245 (top), IMI 133245 (base); IMI 349032 (top); IMI 349032 (base).
